# Pleistocene climate cycling and host plant association shaped the demographic history of the bark beetle *Pityogenes chalcographus*

**DOI:** 10.1038/s41598-018-32617-6

**Published:** 2018-09-21

**Authors:** Martin Schebeck, Eddy J. Dowle, Hannes Schuler, Dimitrios N. Avtzis, Coralie Bertheau, Jeffrey L. Feder, Gregory J. Ragland, Christian Stauffer

**Affiliations:** 1 Department of Forest and Soil Sciences, BOKU, University of Natural Resources and Life Sciences Vienna, Vienna, Austria; 2 Department of Integrative Biology, University of Colorado Denver, Denver, CO USA; 3 Present Address: Department of Anatomy, University of Otago, Dunedin, New Zealand; 4 Department of Biological Sciences, University of Notre Dame, Notre Dame, IN USA; 5 Present Address: Faculty of Science and Technology, Free University of Bozen-Bolzano, Bozen-Bolzano, Italy; 6 Hellenic Agricultural Organisation – Demeter, Forest Research Institute, Thessaloniki, Greece; 7 Laboratoire Chrono-Environnement, Université de Bourgogne Franche-Comté, Pôle Universitaire du Pays de Montbéliard, Montbéliard, France

## Abstract

Historical climatic oscillations and co-evolutionary dependencies were key evolutionary drivers shaping the current population structure of numerous organisms. Here, we present a genome-wide study on the biogeography of the bark beetle *Pityogenes chalcographus*, a common and widespread insect in Eurasia. Using Restriction Associated DNA Sequencing, we studied the population structure of this beetle across a wide part of its western Palaearctic range with the goal of elucidating the role of Pleistocene glacial-interglacial cycling and its close relationship to its main host plant Norway spruce. Genetic distance among geographic sites was generally low, but clustering analysis revealed three genetically distinct groups, that is, southern, central/south-eastern, and north-eastern locations. Thus, three key *P. chalcographus* glacial refugia were identified: in the Italian-Dinaric region, the Carpathians, and the Russian plain, shared with its main host. The current phylogeographic signal was affected by genetic divergence among geographically isolated refugia during glacial periods and postglacial re-establishment of genetic exchange through secondary contact, reflected by admixture among genetic groups. Additionally, certain life history traits, like the beetle’s dispersal and reproductive behaviour, considerably influenced its demographic history. Our results will help to understand the biogeography of other scolytine beetles, especially species with similar life history traits.

## Introduction

Historical events and processes have shaped the distribution and genetic structure of numerous organisms. Past climatic oscillations, like glacial and interglacial cycling during the Pleistocene, were major evolutionary drivers in many species^1,2^. During glacial periods major parts of the northern hemisphere were covered by a thick ice shield. In Europe, glaciation of northern regions and high elevation areas as well as permafrost made these habitats largely inhospitable for life. Glaciation contracted the ranges of many species, geographically restricting populations to glacial refugia^3^. Numerous European species endured glaciation events in the Mediterranean region, though Extra-Mediterranean refugia like the Carpathian Mountains have also been described^3,4^. In many cases, contraction of a species’ range to multiple, isolated refugia limited gene flow and caused genetic divergence among refugia^2^. Following the end of a glacial period, organisms expanded from refugia, recolonizing formerly inhospitable areas and re-establishing genetic exchange. Current population structure thus may reflect past divergence events followed by gene flow during secondary contact^3,5^.

Here, we present a genome-wide study on the biogeography of the bark beetle *Pityogenes chalcographus* (L.) (Coleoptera, Curculionidae, Scolytinae) that provides clear evidence for past genetic divergence in multiple glacial refugia. *Pityogenes chalcographus* has a wide geographic range that now covers major parts of Eurasia, from central Italy to northern Scandinavia and from western Europe to East Asia^6^. The distribution of western Palaearctic *P. chalcographus* is embedded in the range of its main host tree Norway spruce, *Picea abies*^6^, and the range patterns of these two species are likely impacted by climate-driven historical processes^7–10^.

Previous work using single molecular markers describes that European *P. chalcographus* is structured in three geographically distinct mitochondrial clades^7,8^. One clade mainly occurs in northern European regions, another in central Europe, and southern Europe harbours higher haplotype diversity^7,8^. The demography of *P. chalcographus* was likely affected by range changes during the Pleistocene, as these mitochondrial clades diverged at the beginning of the last ice ages about 100,000 years ago^8^. *Pityogenes chalcographus* might have survived the last ice ages in multiple, geographically separated refugia, concordant with those of Norway spruce^7–9^. These refugia were proposed to be located in the Apennines in Italy, in the Carpathians or the Bulgarian Mountains, and in the Russian plain^8^. Moreover, an additional refugium in the Dinaric Alps was suggested^7^.

Other evolutionary drivers resulting in population structure in *P. chalcographus* have been proposed, but with little supporting evidence. For example, heritable bacterial endosymbionts, like *Wolbachia* or *Cardinium*, can alter the reproduction of numerous arthropods, consequently affecting the population structure of their hosts^11,12^. Despite low-titre and low-prevalence endosymbiont infections, the reproductive outcome of *P. chalcographus* is unlikely affected by these bacteria^13,14^.

*Pityogenes chalcographus* is an oligophagous bark beetle, feeding in tree species of a few genera within the plant family Pinaceae^6^, and host specialization has also been proposed to drive population differentiation. Specialization to different host plants results in phenotypic and genetic differentiation among populations of many herbivorous insects^15^. *Pityogenes chalcographus* successfully uses various conifers as hosts in addition to Norway spruce^6^, but does not exhibit host-plant related lineage diversification^16^. Nevertheless, host preference and developmental performance in Norway spruce is highest^17^.

Instead, all available evidence suggests that Pleistocene ice ages were major drivers shaping the biogeography of *P. chalcographus*^8^. Nonetheless, due to the limited resolution of previously applied markers^7,8^ and the complex life history of this species, detailed knowledge on its demographic history, especially during glacial cycling, is scarce. In particular, in-depth information on the number and locality of Pleistocene refugia and impact of postglacial processes on the present genetic structure is lacking. The goal of this study was to make robust inferences about *P. chalcographus* phylogeography using a comprehensive set of genome-wide molecular markers. Studying large panels of single nucleotide polymorphisms (SNPs) provides a powerful approach to inferring phylogeographic history^18,19^. By examining SNP variation among 16 geographic sites spanning a wide part of the *P. chalcographus* range, we provide new insights into glacial-interglacial processes that have shaped the current population structure across geography. We identify key Pleistocene glacial refugia and infer postglacial secondary contact in the context of host plant availability and *P. chalcographus* life history traits. In addition, we discuss species-specific features of *P. chalcographus* and other bark beetles that may influence the inferred phylogeographic patterns. Finally, our study will help to understand demographic processes in other species of the weevil subfamily Scolytinae, especially species with similar life histories.

## Materials and Methods

### Study system

*Pityogenes chalcographus* is a Palaearctic species whose distribution tracks that of suitable host plants. It is oligophagous on various conifers with Norway spruce as the main host in Europe^6^. *Pityogenes chalcographus* preferentially breeds in thin-barked parts of trees, including branches or upper stem sections^20^ and can be a forest pest during environmentally favourable conditions^21^. Male beetles initiate host attacks and attract conspecifics using aggregation pheromones^22^. The species is polygynous; two to seven female beetles mate with one single male, and each female lays on average ten to 26 eggs^23^. After subcortical larval and pupal development, and maturation feeding of young beetles on phloem tissue, a new generation of adults disperses to attack fresh hosts. *Pityogenes chalcographus* can produce up to three generations per year^20^.

### Insect collection, DNA extraction, ddRAD library preparation, and sequencing

**Figure 1:**
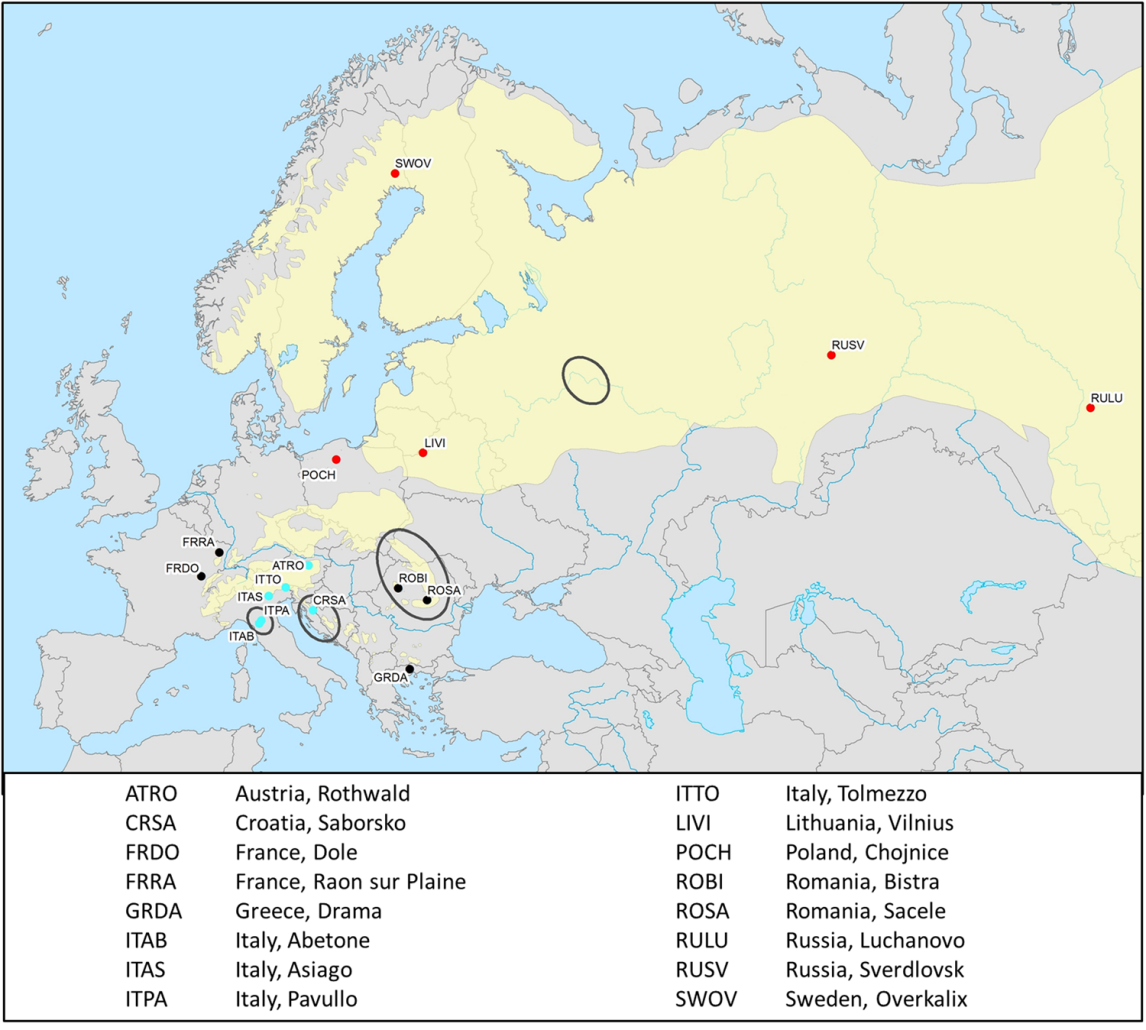
Sample sites of *Pityogenes chalcographus*. Colours refer to assignment to a genetic cluster. Blue: Italian-Dinaric cluster. Red: north-eastern cluster. Black: central/south-eastern cluster. Black circles: key glacial refugia of Norway spruce, *Picea abies*, during the last ice ages. Yellow: present distribution of the primary host Norway spruce.

*Pityogenes chalcographus* was collected from 16 geographic sites between 2004 and 2009 (Fig. 1; Table S1). Living adults were sampled from breeding systems of standing trees or freshly cut logs of Norway spruce and stored in absolute ethanol at −20 °C. To avoid sampling of siblings, only one beetle per breeding system was collected.

DNA was extracted from 192 whole beetles using the ‘GenElute Mammalian Genomic DNA miniprep kit’ (Sigma-Aldrich, St. Louis, MO) following the manufacturer’s instructions. DNA concentrations were estimated using a ‘NanoDrop 2000C’ and all samples were either concentrated or diluted the same concentration ±10% for downstream library preparation.

To study *P. chalcographus* on a multi-locus, genome-wide scale, we applied double digest Restriction Associated DNA Sequencing (ddRADSeq)^24^ using EcoRI and MseI restriction enzymes, as modified from^25^ (details on library preparation and sequencing see SI 2).

### Sequence processing and data filtering

Raw reads were de-multiplexed and filtered as in^18,26^ (for details see SI 3). Due to the lack of a *P. chalcographus* reference genome only forward reads were used in this study. A de-novo ‘pseudo-reference’ genome was created using the *ustacks* algorithm in STACKS (v1.35)^27,28^ (parameters see SI 3). De-multiplexed and filtered reads were mapped to the ‘pseudo-reference’ genome using BWA-MEM (v0.7.12)^29^. SNP calling was performed using the Unified Genotyper in GATK (v3.3.0)^30^. We filtered out SNPs when i) the average base quality-phred score was <20, ii) the total depth per locus across all individuals was <10, iii) sites appeared to be over-assembled (when a chi-square test for the null hypothesis that each allele in a heterozygote has a representation of 0.5 is rejected at p < 0.05), and iv) the minor allele frequency across all individuals was <0.05. Moreover, only individuals with >10,000 retained SNPs were kept for downstream analyses. Location information of SNPs passing these filters was extracted and further analyses were performed in ANGSD (v0.913), using the GATK model^30,31^ (parameters used in ANGSD see SI 3). Individuals with more than 30% missing data were removed and loci that were present in less than 80% of samples were discarded.

### Population statistics

Descriptive statistics and population genetic metrics were inferred from called genotypes calculated in ANGSD, using a final data set with 5,470 loci (requirements for passing filters see above and SI 3). SNPs were converted to a *genind* object in R via the package *adegent* (v2.0.1)^32,33^. Pairwise Nei’s F_ST_ values were calculated in the R package *hierfstat*^34^ and the G”_ST_ value, as an additional, unbiased estimator^35,36^, in the R package *mmod*^37^. Additional population genetic statistics are reported in SI 4.

### Isolation by distance

To assess whether the genetic structure of *P. chalcographus* follows an isolation-by-distance (IBD) pattern a Mantel test^38^, as implemented in the R package *adegenet*^32,33^, was performed. To estimate genetic distances among populations we calculated F_ST_/(1 − F_ST_)^39^ using pairwise Nei’s F_ST_ values as determined above. Geographical distances were inferred from the shortest distance between locations using the R package *geosphere* (v1.5-7)^40^ and were subsequently ln-transformed.

### Relationship and structure among geographic sites

To assess the relationship among *P. chalcographus* geographic sites we used called genotypes as above, converted in a *genpop* object, that is, allele counts per population, in the R package *adegenet*^32,33^. Relationships were calculated applying a NJ-approach with 1,000 bootstrap replicates based on Nei’s distances in the R package *poppr*^41,42^. Results were visualized in FigTree (v1.4.3)^43^.

To study the admixture and population structure of individuals from different locations a structure analysis was performed. NGSadmix^44^ was run using genotype likelihoods, thus considering uncertainty of genotype calls^45^. Genotype likelihoods were calculated in ANGSD and loci were retained when the base quality phred-score was >20, the mapping quality phred-score >20, and the missing data rate was below 30%. Again, only loci present in at least 80% of individuals were retained. This resulted in a total number of 13,105 loci. The optimal number of groups (i.e., K) for clustering individuals was tested for K = 1 to K = 15, using changes in log-likelihood values between Ks^46^, running ten independent replicates with 10,000 iterations each. In addition to the optimal K-value, biologically meaningful Ks, i.e., K = 3 and K = 4, were tested (as previous studies proposed that *P. chalcographus* might have survived Pleistocene glaciation events in either three or four refugia^7,8^). Calculation of optimal K-values and visualization of admixture plots were done in R^47^.

Population structure was further studied by performing a Discriminant Analysis of Principal Components (DAPC), a multivariate statistical method identifying clusters based on linear combinations of genotypes produced by principal component analysis (PCA)^48^. DAPC was calculated in R using the package *adegenet*^32,33,48^ using called genotypes (5,470 loci) as described above. To achieve an optimal discrimination and avoid over-fitting of data, the optimum-α-score to estimate the optimal number of principal components was assessed.

### Coalescent analysis of demography

To test different hypotheses of *P. chalcographus* demography, we performed an approximate Bayesian computation (ABC) analysis implemented in DIYABC (v2.1.0)^49^. We tested four evolutionary scenarios on a reduced data set (only loci with a missing data rate <3% were used, yielding 712 loci; to confirm that the reduced data set is representative a DAPC was performed, SI 5, Fig. S1). Scenario selection was based on results obtained from DAPC, the relationship among geographic sites, and NGSadmix. The following three recent groups (time = 0) were assumed to have originated from an unknown ancestor population (A): Italian-Dinaric (ITDI), north-eastern (NE), and central/south-eastern (CESE). In scenario 1 ITDI and CESE diverged from A in the past (t2) and NE branched off CESE later at t1. In scenario 2 ITDI and NE diverged from A at t2 and CESE branched off NE later at t1. In scenario 3 ITDI and NE originated from A at t2 and merged to CESE at t1. In scenario 4 ITDI, NE, and CESE diverged at the same time (t1) from A (details on DIYABC analysis see SI 5, Table S3).

## Results

In total ~313 million reads were generated with an average of 1.6 million reads per individual. An individual barcode could be assigned to about 81% of raw reads yielding ~1.3 million retained reads per sample. 160 out of 192 individuals passed our filters and were included in the final analyses. Genotype calling yielded a data set with 5,470 SNPs, while 13,105 SNPs passed filters suitable for analysis of genotype likelihoods.

### Population genetics metrics

Based on called genotypes, the average Nei’s pairwise F_ST_ value among geographic sites was 0.039 (details see SI 6, Table S2). The lowest F_ST_ value was observed between the French site FRDO and the Greek site GRDA (=0.021). The highest F_ST_ value was calculated between the Italian site ITTO and the eastern Carpathian site ROSA (=0.071). Further, an overall G”_ST_ value of 0.021 was determined. For additional population statistics see SI 4, Table S2.

### Isolation by distance

**Figure 2:**
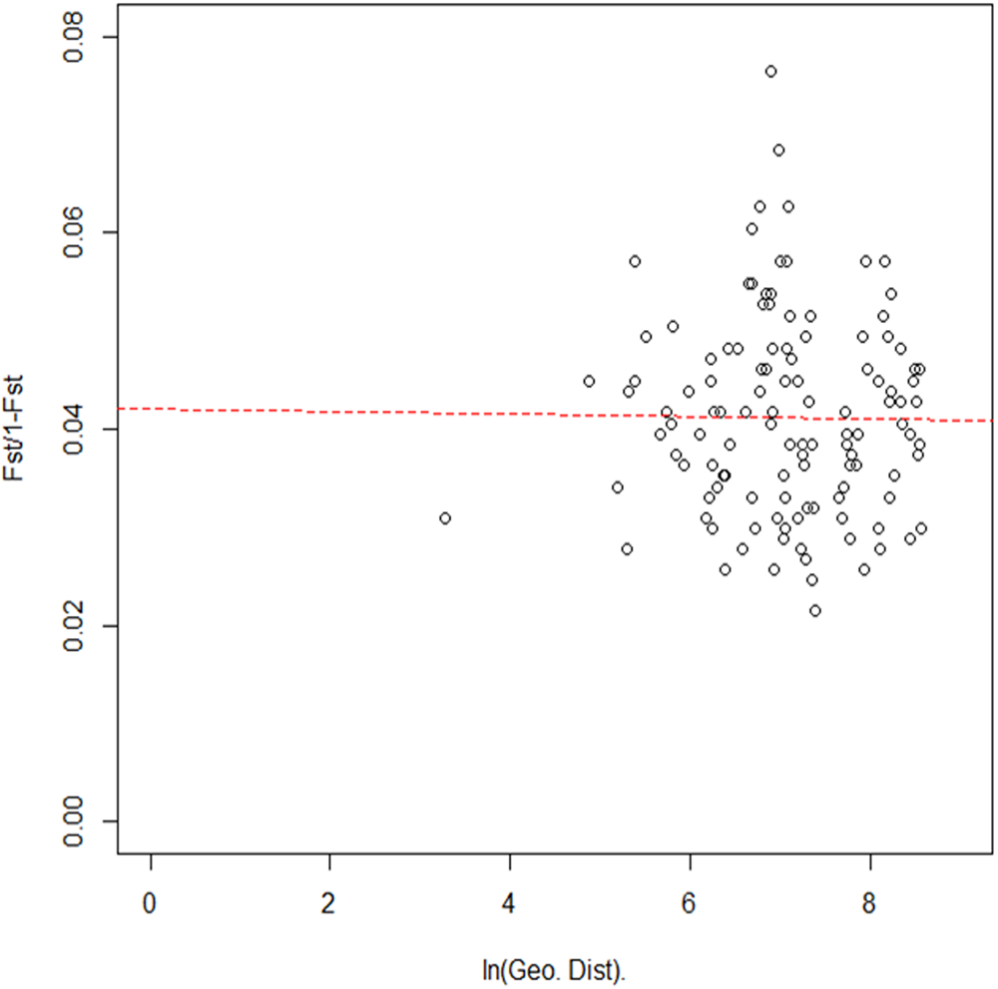
Mantel test for isolation by distance of *Pityogenes chalcographus* using Nei’s pairwise F_ST_ values and ln-transformed geographic distances (5,470 loci analysed).

The genetic structure of *P. chalcographus* does not follow a strong IBD-pattern. The Mantel test did not reveal a significant positive correlation between genetic and geographic distances among locations (r Mantel = −0.011, p = 0.534, Fig. 2).

### Relationships among geographic sites

**Figure 3:**
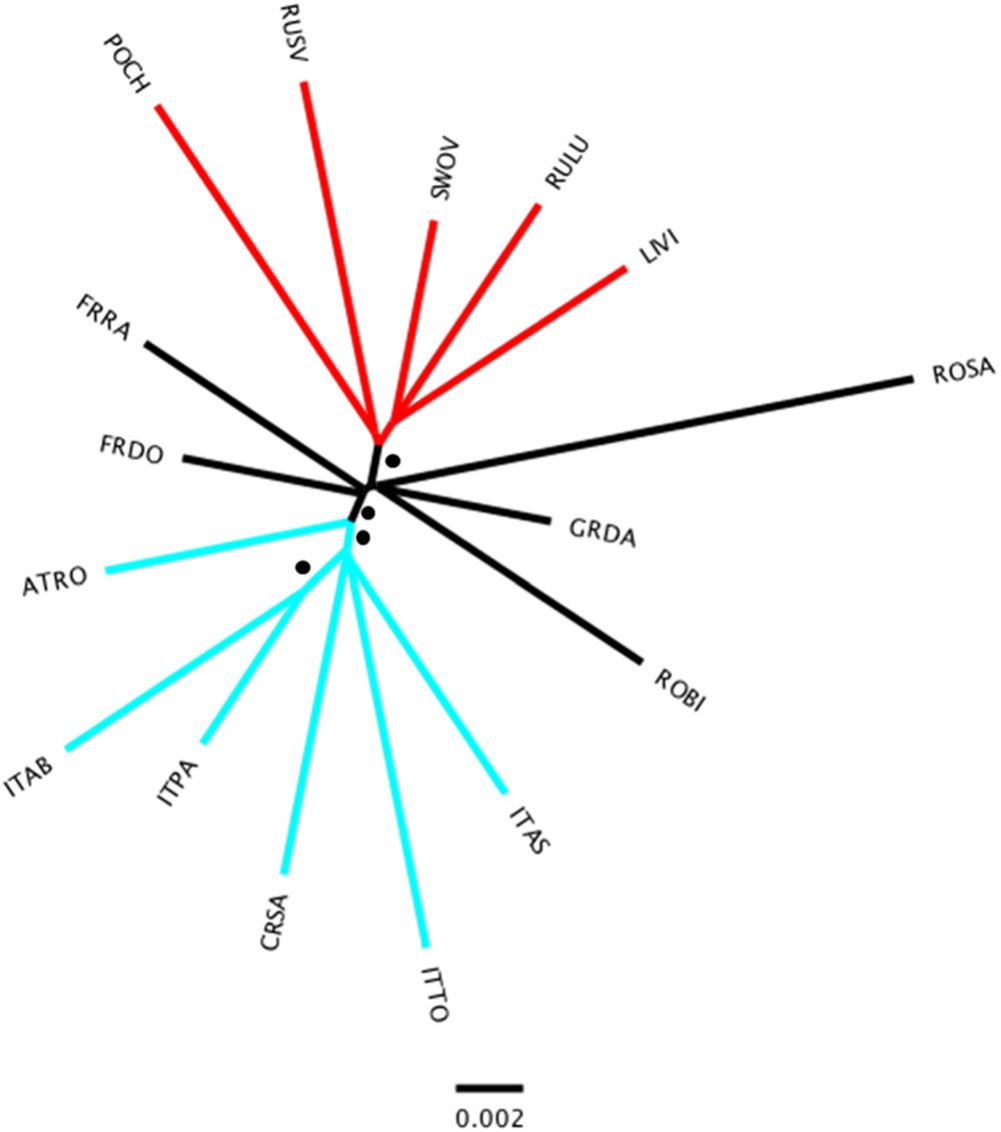
Neighbour Joining tree among *Pityogenes chalcographus* geographic sites based on Nei’s distances using 1,000 bootstrap replicates, using called genotypes (5,470 loci analysed; abbreviations of geographic sites see Fig. 1). Blue: Italian-Dinaric cluster. Red: north-eastern cluster. Black: central/south-eastern cluster. • bootstrap value = 100.

The NJ-tree based on Nei’s distances among geographic sites revealed a low level of genetic differentiation, reflected by relatively short branch lengths (Fig. 3). The average Nei’s distance was 0.020 with a maximum value of 0.033 between the Italian site ITTO and the Carpathian site ROSA. The minimum (=0.011) was calculated between the Greek location GRDA and the French site FRDO. Nonetheless, sites were largely grouped by geography with clusters consistent with proposed glacial refugia. Southern European *P. chalcographus* from Italian-Dinaric locations (CRSA, ITAB, ITAS, ITPA, ITTO; Fig. 3 blue) group together, supported by a bootstrap value of 100, and the Austrian site (ATRO) is sister to this Italian-Dinaric cluster (bootstrap value = 100). Within the Italian-Dinaric cluster, the two Apennine sites (ITAB, ITPA) are also supported by a bootstrap value of 100. Further, the north-eastern sites from Europe and Asian Russia (LIVI, POCH, RULU, RUSV, SWOV; Fig. 3 red) form one well-supported cluster (bootstrap value = 100). The remaining central/south-eastern European sites (FRDO, FRRA, GRDA, ROBI, ROSA; Fig. 3 black) did not form a distinct geographic cluster.

### Admixture and structure among individuals

**Figure 4:**
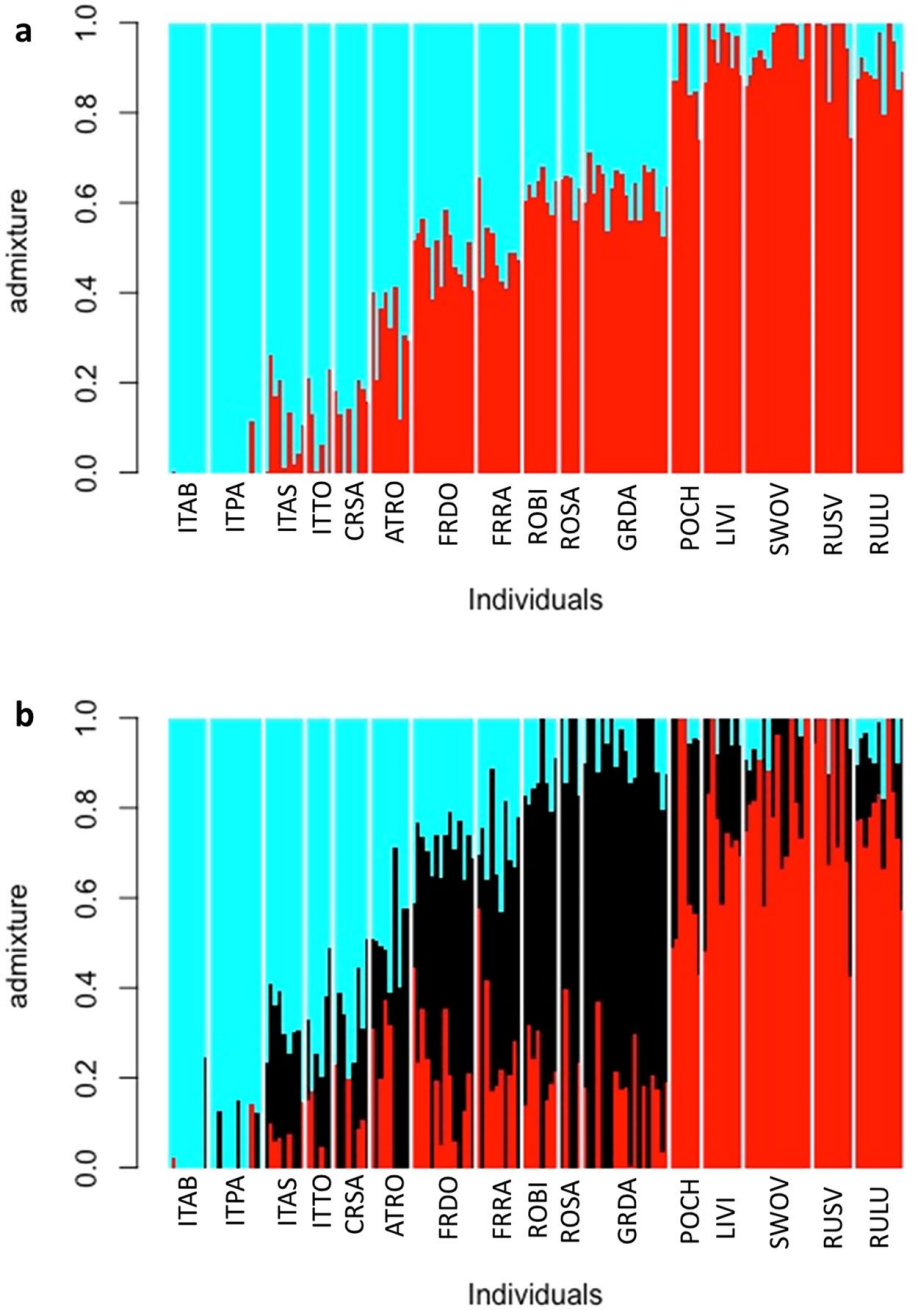
Admixture plot (NGSadmix) for K = 2 (**a**) and K = 3 (**b**) for *Pityogenes chalcographus*, using genotype likelihoods (13,105 loci analysed; abbreviations of geographic sites see Fig. 1).

NGSadmix analysis resulted in an optimal K = 2 (SI 7, Fig. S2), also suggesting two distinct geographic clusters. One clear cluster covered individuals from north-eastern sites (LIVI, POCH, RULU, RUSV, SWOV; Fig. 4a red). Individuals sampled from these locations showed relatively low amounts of admixture. The second distinct cluster comprised samples from the Italian-Dinaric region (CRSA, ITAB, ITAS, ITPA, ITTO; Fig. 4a blue). Within the Italian-Dinaric cluster (blue), the two northern Italian sites (southern slopes of the Alps; ITAS, ITTO) and the Dinaric location (CRSA) had higher amounts of admixture than the southernmost Italian sites from the Apennines (ITAB, ITPA). Samples belonging to the central/south-eastern European sites had a higher amount of admixture and could not clearly be assigned to one of the two clusters. In contrast to the other central/south-eastern sites (FRDO, FRRA, GRDA, ROBI, ROSA), the highly admixed Austrian location (ATRO) had a greater proportion of the Italian-Dinaric (blue) cluster than of the north-eastern (red) cluster.

NGSadmix analysis with K = 3, which would be consistent with hypothesized three key glacial refugia of *P. chalcographus*^8^, again supported the same two, relatively cohesive, geographic clusters in the north-eastern (LIVI, POCH, RULU, RUSV, SWOV; Fig. 4b red) and the Italian-Dinaric regions (CRSA, ITAB, ITAS, ITPA, ITTO; Fig. 4b blue). Again, the Austrian site (ATRO) was highly admixed but its major cluster is from the Italian-Dinaric region (blue). The third cluster (black) was relatively diffuse across geography; the remaining central/south-eastern populations (FRDO, FRRA, GRDA, ROBI, ROSA) did not form a clear cluster as was the case for K = 2 suggesting high levels of admixture. In the north-eastern cluster, the three locations furthest northeast (RULU, RUSV, SWOV) demonstrated relatively low admixture, as did the two southernmost from the Apennines (ITAB, ITPA). Admixture in other north-eastern and remaining Italian-Dinaric sites was more extensive.

Structure analysis with K = 4, consistent with an alternative hypothesis of four glacial refugia for *P. chalcographus*^7^, yielded qualitatively similar results to the K = 3 analysis (SI 8, Fig. S3).

### Grouping of individuals across geography

**Figure 5:**
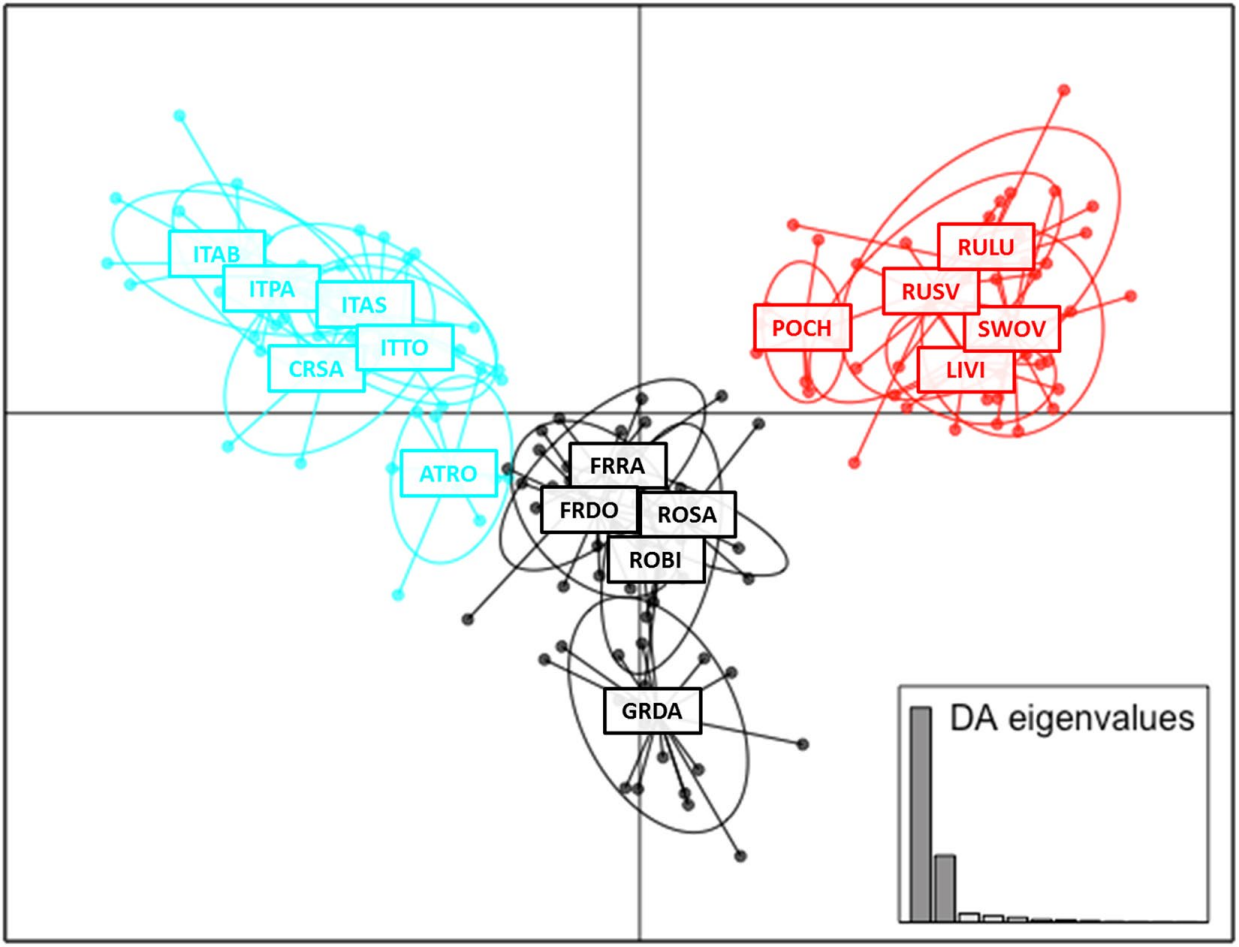
Grouping of *Pityogenes chalcographus* individuals across geography, using a Discriminant Analysis of Principal Components (DAPC) and called genotypes (5,470 loci analysed; abbreviations of geographic sites see Fig. 1). Blue: Italian-Dinaric group. Red: north-eastern group. Black: central/south-eastern group.

DAPC results provide support for three distinct, geographic *P. chalcographus* groups (Fig. 5). Again, one group comprised north-eastern sites (LIVI, POCH, RULU, RUSV, SWOV; Fig. 5 red). Individuals from this group were clearly separated from the other locations. A second group included the Italian-Dinaric sites and the Austrian location (ATRO, CRSA, ITAB, ITAS, ITPA, ITTO; Fig. 5 blue). Individuals from this group did not overlap with the north-eastern group and had a very limited overlap with the remaining central/south-eastern group (Fig. 5 black). Individuals from the Italian-Dinaric sites (CRSA, ITAB, ITAS, ITPA, ITTO) had no overlap with the central/south-eastern group, only the Austrian location (ATRO) had a slight overlap with the central site in France (FRDO). Although being highly admixed, the remaining central/south-eastern sites (FRDO, FRRA, GRDA, ROBI, ROSA) were found to form an additional group. Apart from the French location mentioned above, they did not overlap with other groups (Fig. 5).

Additionally, distances on the DAPC axes reflect finer-scale geographic information: For example, the Italian-Dinaric sites, including the Austrian location, are closer to the remaining central/south-eastern sites than to the north-eastern sites. Within the central/south-eastern group (black), the Greek site GRDA is the south-eastern-most and closest to the two Carpathian sites (ROSA, ROBI). The Austrian location, for example, is close to the northern Italian sites (ITAS, ITTO), and to the two central sites in France (FRDO, FRRA). The Apennine sites ITAB and ITPA are the southernmost and therefore on the edge of the Italian-Dinaric (blue) group. Within the north-eastern group (red), the Polish location POCH is closest to the central/south-eastern group (Fig. 5).

### Demographic history

**Figure 6:**
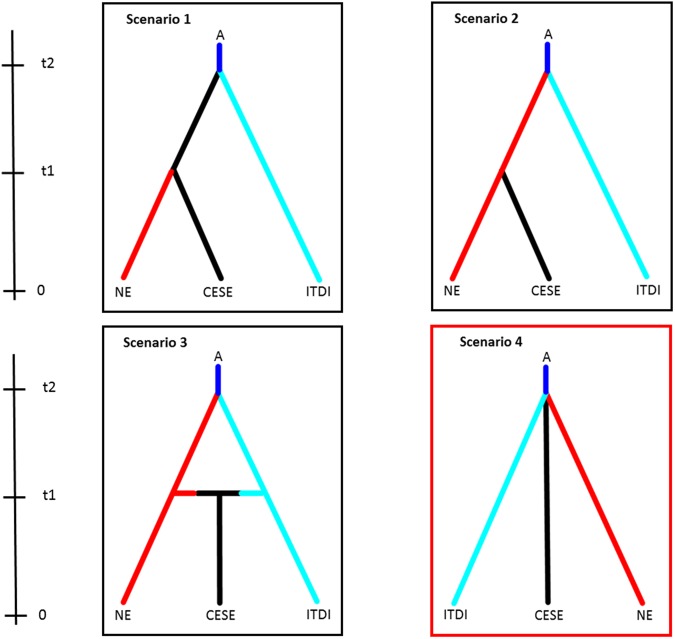
Evolutionary scenarios to describe the demographic history of *Pityogenes chalcographus* using approximate Bayesian computation (ABC) analysis on a reduced data set (712 loci). A (ancestral population), CESE (central/south-eastern sites), NE (north-eastern sites), ITDI (Italian-Dinaric sites). Scenario 4 was best supported (red frame). Posterior probability (PP) scenario 1 = 0.0076, 95% CI 0.0060–0.0092; PP scenario 2 = 0.0066, 95% CI 0.0052–0.0079; PP scenario 3 = 0.0012, 95% CI 0.0009–0.0014, PP scenario 4 = 0.9847, 95% CI 0.9821–0.9873.

The best-supported scenario describing the demographic history of *P. chalcographus* was scenario 4 (Fig. 6). The posterior probability (PP) of scenario 4 was 0.9847 (95% CI 0.9821–0.9873) and was higher than the posterior probabilities of the other three scenarios (PP scenario 1 = 0.0076, 95% CI 0.0060–0.0092; PP scenario 2 = 0.0066, 95% CI 0.0052–0.0079; PP scenario 3 = 0.0012, 95% CI 0.0009–0.0014). This suggests that the Italian-Dinaric (ITDI), north-eastern (NE), and central/south-eastern (CESE) groups most probably diverged from an unknown ancestral population (A) at the same time point. The type I error for scenario 4 was 0.058 (i.e., probability that another scenario is chosen although scenario 4 is correct), the type II error for this scenario was 0.089 (i.e., probability that this scenario is chosen although it is not the correct one).

## Discussion

We present a genome-wide, biogeographic analysis of the bark beetle *P. chalcographus* with emphasis on the influence of Pleistocene climatic oscillations on its present population structure. Analyses of clustering, relationships of geographic sites as well as a Bayesian approach testing population ancestry provide evidence for past contraction into Pleistocene glacial refugia and NGSadmix shows subsequent population admixture. Our data suggest a grouping of current *P. chalcographus* populations into three main, geographic clusters: one cluster comprising north-eastern sites, a second cluster covering Italian-Dinaric locations and a central European site from Austria, and a third cluster consisting of other central/south-eastern European sites. The genetic structure of *P. chalcographus* is characterized by high levels of admixture and low levels of differentiation among geographic sites. Pleistocene climatic events and certain species-specific traits were likely the main drivers that shaped the genetic architecture of *P. chalcographus*. Furthermore, the combination of our phylogeographic data and that of Norway spruce^9^ allows for the re-evaluation of the locality and number of glacial refugia and to assess the importance of secondary contact during postglacial expansion in this important bark beetle.

Past climatic fluctuations resulted in range changes of many organisms, shaping much of their present day genetic structure^3^. Demographic processes of certain insect groups were additionally affected by co-evolutionary dependencies. Bark beetles belong to the species-rich family Curculionidae, a group with long-standing plant-associations^50^. As most bark beetle life-cycle events occur in plant tissues, the geographic range of these insects is tightly associated with the geographic range of their main hosts^8,18,51–53^. *Pityogenes chalcographus* is oligophagous on conifers^6^. Although this beetle shows no host-related lineage diversification^16^, Norway spruce is favoured over other tree species^17^. Further, present range overlaps of European *P. chalcographus* are most consistent with Norway spruce^6^. Thus, we hypothesize that this insect-plant relationship resulted in range contraction of both, the host tree and the beetle, during glaciation events in the Pleistocene.

Cold conditions during Pleistocene glaciation forced Norway spruce to retreat to multiple refugia. Supported by pollen fossils and molecular data, these key refugia were located in the Russian plain, the Carpathians/Bulgarian Mountains, the Apennines, and the Dinaric Alps^9,10,54,55^. The refugium in the Apennines, however, is contentious^56^. In addition, numerous small refugia in Europe have been described^9^.

Judging by single molecular markers, early events (about 100,000 years BP) during the last ice ages caused divergence of mitochondrial clades^8^ and up to four *P. chalcographus* glacial refugia were hypothesized, consistent with those of Norway spruce^7,8^. Our analyses provide comprehensive insights in Pleistocene contraction-expansion processes of *P. chalcographus* to infer the number and locality of refugia, and assess subsequent postglacial admixture. DAPC results and the NJ-tree suggest two distinct genetic clusters and one diffuse genetic cluster, and the ABC analysis clearly supports a scenario with three clusters originating from a single, ancestral population. Moreover, these results suggest that current *P. chalcographus* populations clearly cluster by geography. Taking these results together and considering data on the Pleistocene history of Norway spruce, we suggest three *P. chalcographus* glacial refugia in the western Palaearctic.

Clustering of *P. chalcographus* from Russian, Swedish, Polish, and Lithuanian sites (north-eastern group) indicates that these individuals survived periods of increased glaciation in the Russian plain. Grouping of beetles from the French, Romanian, and Greek sites (central/south-eastern group) suggests a refugium in the Carpathians (or Bulgarian Mountains)^9,10^. Italian-Dinaric *P. chalcographus*, including beetles from the Austrian site, might have survived glaciation events in southern Europe, where major Norway spruce refugia in the Apennines and the Dinaric Alps were found^10,55^. These two mountain ranges are geographically relatively close and several small refugia between them have been reported^57^. Because *P. chalcographus* is a long-range disperser^58^, we propose one Italian-Dinaric *P. chalcographus* refugium.

In addition to these refugia, *P. chalcographus* might have survived Pleistocene ice ages also in the more eastern part of its range. Due to its oligophagous feeding behaviour, it can utilize Asian conifers, like *Picea obovata*, *Picea jezoensis*, or *Pinus koraiensis*, as hosts^6^. Several refugia of these tree species have been described in Asia, for example in Siberia (*P. obovata*)^59^ or in the Far East (*P. koraiensis*^60^, *P. jezoensis*^61^). Thus, *P. chalcographus* might have endured unfavourable conditions during the Pleistocene even in these regions, shared with a respective host. However, detailed knowledge on the genetic population structure of *P. chalcographus* from the eastern Palaearctic and its influence on European populations are not available and future research should focus on extensive sampling of this region to get a more complete picture of the beetle’s biogeography.

After the end of the last ice ages about 10,000 years ago, *P. chalcographus* expanded its range to previously inhospitable habitats. As for glacial periods, we assume that recolonization of the beetle and its main host also occurred parallel. For example, present Norway spruce in Fennoscandia was recolonized from a single refugium in the Russian plain^9,62,63^. Clustering of *P. chalcographus* from north-eastern locations suggests that these beetles also originated predominately from this Russian refugium, consistent with a tight insect-host relationship. Assuming three *P. chalcographus* refugia, NGSadmix results suggest secondary contact, for example, in Poland where north-eastern and central/south-eastern European beetles admixed (Fig. 4). We cannot rule out the possibility of shared polymorphisms among refugia contributing to the genetic clustering results. However, the geographic areas with the greatest inferred admixture for the beetles were also a secondary contact zones for Norway spruce^62^, again supporting a shared contraction and expansion history of bark beetle and host tree. *Pityogenes chalcographus* from central/south-eastern European sites show high amounts of admixture. These beetles might predominantly have expanded from a refugium in the Carpathians with individuals from sites in relative close proximity to this mountain range (ROBI, ROSA, GRDA) having lower amounts of admixture. Moreover, NGSadmix results suggest that *P. chalcographus* from this central European refugium also contributed to the genetic architecture of north-eastern European and northern Italian locations, reflecting additional secondary contact, for example, in the Southern Alps and Austria.

Recolonization from the Italian-Dinaric refugium mainly accounted for the genetic structure of southern sites. The southernmost populations in this area (ITAB, ITPA, CRSA) show low amounts of admixture compared to the northern Italian sites and the Austrian location. Furthermore, NGSadmix results suggest that *P. chalcographus* from the Italian-Dinaric refugium may also have contributed to the genetic structure of central/south-eastern sites and only limited to north-eastern sites.

Similar biogeographic patterns, that is, climate-driven range contractions and expansions and a tight host plant association, have also been reported from other insect species. For example, numerous butterflies (Lepidoptera) are characterized by their close relationship to plants, resulting in mutual adaptations to each other and overlapping distribution areas of insect and host^64^. Furthermore, various European lepidopteran species show demographic patterns affected by climatic fluctuations during the Pleistocene similar to *P. chalcographus*: range contractions to geographically isolated refugia in Mediterranean and Extra-Mediterranean regions resulted in divergence among these refugia, followed by postglacial secondary contact and re-establishment of genetic exchange^2,65–67^. In addition, diversification of the butterfly family Nymphalidae was found as a result of angiosperm radiation during the Cretaceous era^68^, a comparable co-evolutionary history as reported for weevils^50^. Taking this knowledge together underlines our hypothesis that glacial-interglacial cycling and the association to its main host plant were major evolutionary drivers in *P. chalcographus*.

In addition to the influence of past climatic oscillations, specific life history traits likely contributed to the current genetic structure of *P. chalcographus*. One important trait that affects the genetic structure of bark beetles is their dispersal behaviour^18^. IBD describes an increase of genetic differentiation among sites with increasing geographic distance^69^, presumably because of limited dispersal^70^. *Pityogenes chalcographus* can spread over long distances, more than 80 km aided by wind^58^, facilitating exchange of genetic material among sites. This feature might explain the absence of an IBD pattern in *P. chalcographus*, as long-range dispersing insects are generally characterized by a weak relationship between genetic and geographic distance^71^. In addition to the beetle’s intrinsic, species-specific traits, anthropogenic influence might also explain the lack of IBD. Various insects, like the bark beetle *Tomicus piniperda*^72^, the fire ant *Solenopsis invicta*^73^, or the leafhopper *Scaphoideus titanus*^74^ exhibit an absence of this pattern, for example, due to human trade. As humans have been trading across Eurasia for more than 2000 years^75,76^, we therefore cannot rule out a human influence on the genetic structure of *P. chalcographus*.

Further, the main host of *P. chalcographus*, Norway spruce, has a wide, current range^10^ and was also very common before the last ice ages^77,78^. The absence of insuperable physical barriers in the ranges of *P. chalcographus* and its main host^9^ probably results the low level of population differentiation and high amounts of admixture found in this study. This is corroborated by the relatively close relationship of *P. chalcographus* from Italian-Dinaric and the Austrian sites, suggesting that the Alps were not a strong barrier for dispersal of these two species. In contrast, physical barriers were found to affect the genetic architecture of other bark beetles, for example the mountain pine beetle *Dendroctonus ponderosae* in North America, where deserts and mountain ranges hampered gene flow among populations^18,52,53^.

Another factor that might have influenced the genetic architecture of *P. chalcographus* is its reproductive behaviour. It releases aggregation pheromones, a complex system of semiochemicals^22^, to attract male and female conspecifics. It is polygynous where one male can mate with several females, has a high fecundity, and can produce up to three generations per year^20,23^. *Pityogenes chalcographus* might have established thousands of generations of different origin since the end of the last glaciation events, likely reflected in high levels of admixture and low levels of differentiation.

In conclusion, the phylogeography of *P. chalcographus* was shaped by past climatic oscillations during the Pleistocene and co-evolutionary processes with its main host plant. Limited exchange of genetic material among different refugia during glaciation events is still reflected in the phylogeographic signal observed today; however, extensive postglacial admixture as a result of certain intrinsic traits has resulted in low levels of differentiation.

## Electronic supplementary material

Supplementary Information

## Data Availability

Sequence data will be provided upon request.
