# Supplementary material for: Pleistocene climate cycling and host plant association shaped the demographic history of the bark beetle Pityogenes chalcographus
Source: Sci Rep. 2018 Sep 21;8:14207. doi: 10.1038/s41598-018-32617-6 (PMC6155062; doi:10.1038/s41598-018-32617-6)
Supplement: Supplementary file 1 — Supplementary Information [file 41598_2018_32617_MOESM1_ESM.pdf]

<u>Supplementary Information</u>

Pleistocene climate cycling and host plant association shaped the demographic history of the bark beetle *Pityogenes chalcographus*

Authors

Martin Schebeck, Eddy J. Dowle, Hannes Schuler, Dimitrios N. Avtzis, Coralie Bertheau, Jeffrey L. Feder, Gregory J. Ragland, Christian Stauffer

Supplementary Information SI 1 – Overview on analysed individuals

Table S1. Location information (name, coordinates, abbreviations), year of collection, and sample sizes post-filtering (N) of *Pityogenes chalcographus*

Site	Coordinates	Abbreviation/ Map code	Year of collection	N
Austria/Rothwald	47°45′ N/15°04′ E	ATRO	2004	9
Croatia/Saborsko	44°59′ N/15°28′ E	CRSA	2009	8
France/Dole	47°05′ N/05°29′ E	FRDO	2007	14
France/Raon sur Plaine	48°31' N/07°06' E	FRRA	2004	10
Greece/Drama	41°08′ N/24°09′ E	GRDA	2004	19
Italy/Abetone	44°08′ N/10°39′ E	ITAB	2009	9
Italy/Asiago	45°52' N/11°30' E	ITAS	2004	9
Italy/Pavullo	44°20' N/10°50' E	ITPA	2004	12
Italy/Tolmezzo	46°24' N/13°01' E	ITTO	2004	6
Lithuania/Vilnius	54°04′ N/25°20′ E	LIVI	2004	9
Poland/Chojnice	53°42' N/17°34' E	POCH	2004	7
Romania/Bistra	46°22′ N/23°06′ E	ROBI	2004	8
Romania/Sacele	45°37' N/25°42' E	ROSA	2004	5
Russia/Luchanovo	56°21' N/85°02'	RULU	2009	11
Russia/Sverdlovsk	58°53′ N/61°51′ E	RUSV	2009	9
Sweden/Overkalix	66°19′ N/22°50′ E	SWOV	2004	15

Supplementary Information SI 2 – library preparation and sequencing

ddRAD library preparation and sequencing

For analyzing the demographic history of *P. chalcographus* on a multi-locus and genome-wide level, double digest Restriction Associated DNA Sequencing (ddRADSeq) was applied. ddRAD libraries were prepared after [1], as modified after [2]. 200 ng of genomic DNA per sample were digested using the restriction enzymes EcoRI and MseI. Afterwards, barcoded (8 to 10 nucleotides long as used in [2]) EcoRI and non-barcoded MseI adapters were ligated to the digested DNA fragments. Restriction-digested and adapter-ligated fragments were PCR-amplified (30 cycles) with primers complementary to the adapters. Afterwards, PCR products of all samples were pooled and purified using magnetic beads (Agencourt AMPupre XP, Beckman Coulter, Indianapolis, IN). Pools were size-selected for a fragment length of 400-500 bp using a BluePippin System (automated DNA size selection system, Sage Science, Beverly, MA). Quality control of libraries was performed on a 'Bioanalyzer 2100' (Agilent) and a 'Qubit 2.0 Fluorometer' (Invitrogen, Inc.). The library of 192 samples was paired-end-sequenced with 100 bp reads on a single Illumina HiSeq2000 lane (BGI Americas, Cambridge, MA).

Supplementary Information SI 3 – data processing

De-multiplexing and filtering

De-multiplexing and filtering of raw reads was done using a python script by [3], as used in [4], allowing one barcode and one cut-site mismatch, and retaining only reads longer than 20 bp.

Creating the 'pseudo reference' genome

The 'pseudo-reference' genome was created applying *ustacks* in STACKS (v1.35) [5,6]. Following parameters were used: minimum depth to create a stack = 3, maximum distance between stacks = 2, retain unused reads, disabling haplotype calls from secondary reads, and enabling the deleveraging algorithm for over merged stacks. Only *consensus* contigs with log-likelihood values >-20 and >10 mapped reads were retained for further analyses.

Genotype calling

After SNP calling in GATK using the Unified Genotyper, SNP location was extracted and further analysis were performed in ANGDS(v0.913) [7] using the GATK model. The following filters on reads and SNPs were applied in ANGSD to retain high-quality called genotypes: a base quality phred-score >20, a mapping quality phred-score >20, a polymorphic p-value <1e-6, and a SNP posterior cut-off >0.95.

Supplementary Information SI 4 – population statistics

Additional population statistics metrics

Population statistics metrics were inferred from called genotypes using 5,470 sites, calculated in ANGSD. Testing for Hardy-Weinberg-equilibrium (R function *hw.test*) and calculation of theta (R function *theta.k*) [8] were performed in the R package *pegas* [9]. Tajima's D [10] was calculated with the R package *strataG* (R function *tajimasD*) [11]. Observed and expected heterozygosity, genotypic richness, genotypic diversity and genotypic evenness were calculated using the R package *poppr* [12,13].

No significant deviations from Hardy-Weinberg-equilibrium were found ($p = 0.343$), theta was 0.186. Overall Tajima's D was -0.061 ($p = 0.499$). Overall, observed heterozygosity (= 0.189) was smaller than expected heterozygosity (= 0.207).

Results on genotypic richness, genotypic diversity, and genotypic evenness see Table S2.

Table S2. Overview on *Pityogenes chalcographus* genotypic richness (multilocus genotypes), genotypic diversity (Simpon's index), and genotypic eveness per population (abbreviations of geographic sites see Fig. 1 and Table S1).

Site	Genotypic richness (multilocus genotypes	Genotypic diversity (Simpson's index)	Genotypic evenness
ITAS	9	0.889	1
ITTO	6	0.883	1
ATRO	9	0.889	1
ITPA	12	0.917	1
FRRA	10	0.9	1
ROSA	5	0.8	1
ROBI	8	0.875	1
POCH	7	0.857	1
SWOV	15	0.933	1
GRDA	19	0.947	1
ITAB	9	0.889	1
FRDO	14	0.929	1
CRSA	8	0.875	1
LIVI	9	0.889	1
RULU	11	0.909	1
RUSV	9	0.889	1

Supplementary Information SI 5 – approximate Bayesian computing (ABC) to analyze the demography of *P. chalcographus*

Four evolutionary scenarios for the demographic history of *P. chalcographus* were tested, using ABC analysis implemented in DIYABC 2.1.0 [14]. For each of the four scenarios 1 million simulations and 36 summary statistics were calculated, assuming uniform prior distributions (details on prior parameters see Table S3). To estimate posterior probabilities for each scenario via logistic regression and to assess posterior parameters for the best-supported scenario, the closest 1% of the simulated data was used. Confidence parameters of the selected scenario were estimated via type I and type II errors from 500 pseudo-observed data sets.

Table S3. Scenario parameters for ABC analysis of *Pityogenes chalcographus* in DIYABC 2.1.0 (Cornuet et al. 2014). A (ancestral population), CESE (central/south-eastern sites), NE (north-eastern sites), ITDI (Italian-Dinaric sites).

Parameter	Prior distribution	Minimum prior value	Maximum prior value
Population size (ITDI)	Uniform	10	300,000
Population size (CESE)	Uniform	10	300,000
Population size (NE)	Uniform	10	300,000
Population size (A)	Uniform	10	500,000
Time (t1)	Uniform	1,000	3,000,000
Time (t2)	Uniform	1,000	3,000,000
Admixture rate (ra)	Uniform	0.001	0.999

The four evolutionary scenarios were tested on a reduced data set. To confirm that this data yield similar results a DAPC was performed (Fig. S1).

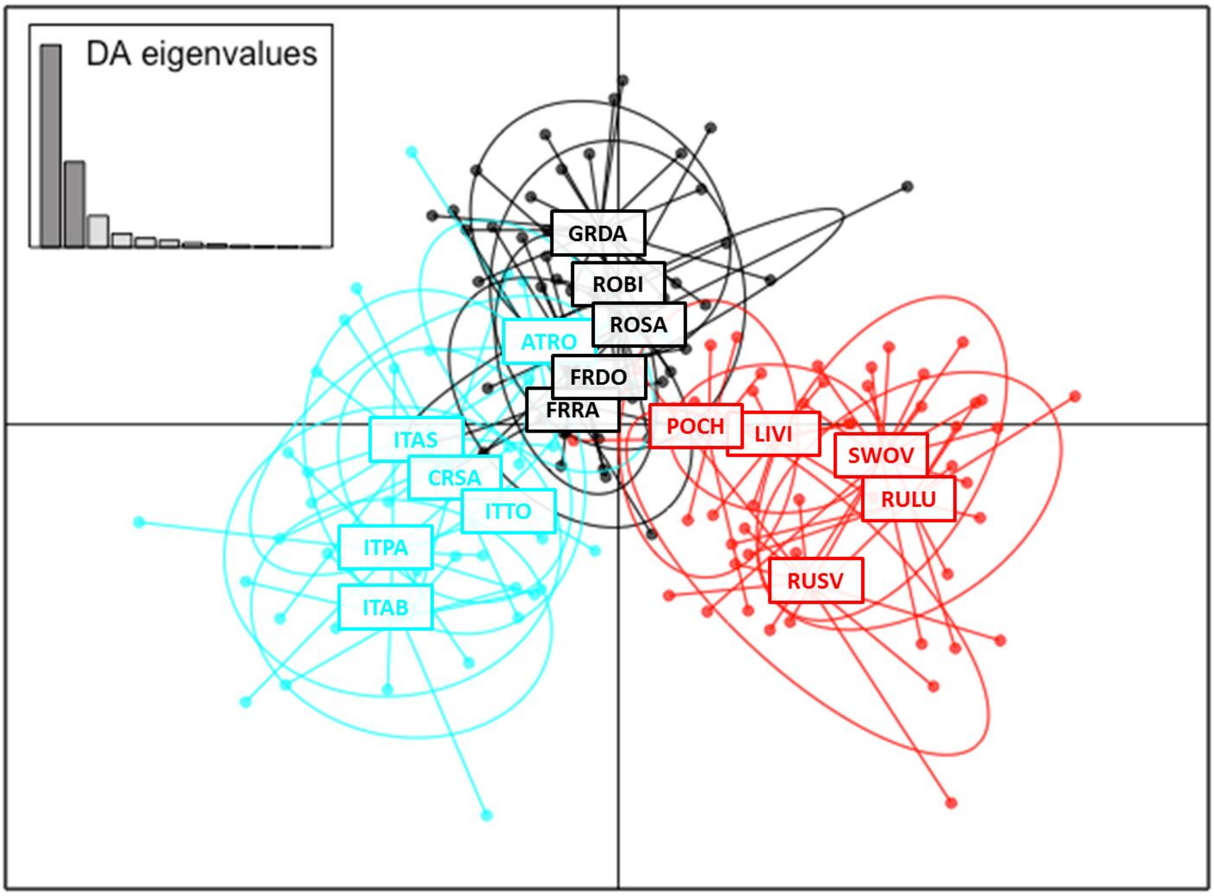

Figure S1. DAPC of a reduced data set analyzing 712 loci of *Pityogenes chalcographus* for use in DIYABC 2.1.0. Abbreviations of geographic sites see Fig. 1 and Table S1.

Supplementary Information SI 6 – details on pairwise Nei's F_{ST} values

Using called genotypes (5,470 loci) Nei's pairwise F_{ST} value among geographic sites were calculated using the R package *hierfstat* [15].

Table S4. Pairwise Nei's F_{ST} values among *Pityogenes chalcographus* geographic sites (abbreviations of geographic sites see Fig. 1 and Table S1).

	ATRO	CRSA	FRDO	FRRA	GRDA	ITAB	ITAS	ITPA	ITTO	LIVI	POCH	ROBI	ROSA	RULU	RUSV
CRSA	0,040														
FRDO	0,027	0,032													
FRRA	0,034	0,040	0,027												
GRDA	0,025	0,029	0,021	0,024											
ITAB	0,040	0,042	0,035	0,040	0,032										
ITAS	0,036	0,039	0,030	0,038	0,029	0,042									
ITPA	0,032	0,035	0,029	0,033	0,028	0,030	0,033								
ITTO	0,043	0,047	0,034	0,043	0,030	0,048	0,043	0,038							
LIVI	0,040	0,045	0,031	0,036	0,026	0,049	0,043	0,041	0,049						
POCH	0,046	0,051	0,034	0,044	0,031	0,054	0,050	0,043	0,059	0,045					
ROBI	0,037	0,046	0,030	0,037	0,025	0,046	0,044	0,039	0,052	0,042	0,050				
ROSA	0,052	0,057	0,037	0,047	0,029	0,059	0,054	0,046	0,071	0,051	0,064	0,054			
RULU	0,038	0,043	0,029	0,036	0,028	0,044	0,041	0,037	0,044	0,032	0,041	0,039	0,046		
RUSV	0,043	0,049	0,034	0,041	0,029	0,051	0,047	0,042	0,054	0,040	0,047	0,044	0,054	0,037	
SWOV	0,032	0,036	0,028	0,030	0,025	0,038	0,035	0,035	0,037	0,027	0,035	0,033	0,038	0,027	0,032

Supplementary Information SI 7 – details on NGSadmix analyses

Results for the optimal number of Ks inferred from genotype likelihoods (analysing 13,105 loci) and changes in log-likelihood values between Ks [16], tested for K1 to K15.

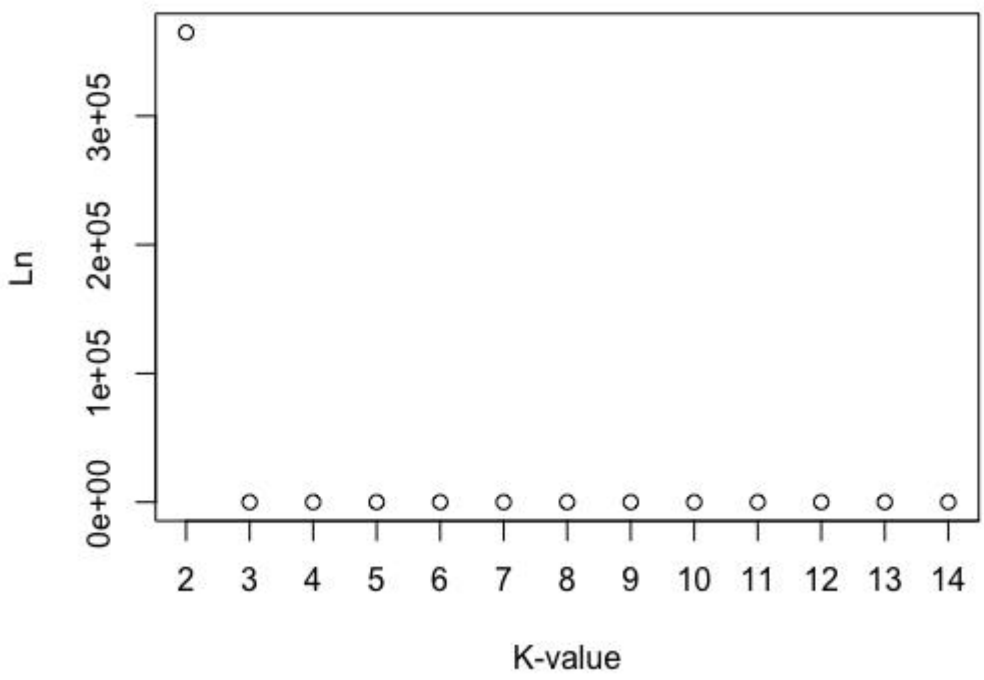

Figure S2. Optimal K-value (=2) based on genotype likelihoods (analysing 13,105 loci) and changes in log-likelihood values.

Supplementary Information SI 8 – details on NGSadmix analyses

In addition to K =2 and K = 3, NGSadmix analysis for K = 4 was performed (Fig. S3).

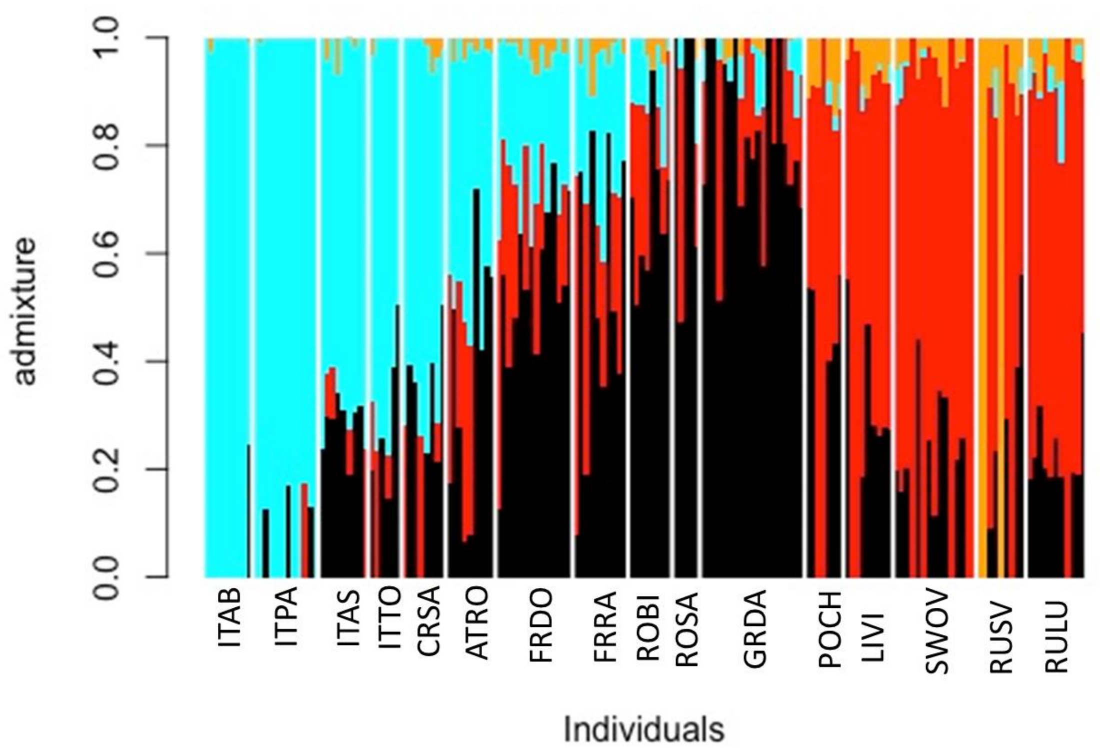

Figure S3. Admixture plot (NGSadmix) for K = 4 for *Pityogenes chalcographus*, using genotype likelihoods (13,105 loci analysed, abbreviations of geographic sites see Fig. 1 and Table S1).

References

1 Parchman, T. L. *et al.* Genome-wide association genetics of an adaptive trait in lodgepole pine. *Mol Ecol* **21**, 2991-3005, doi: 10.1111/j.1365-294X.2012.05513.x (2012).

2 Egan, S. P. *et al.* Experimental evidence of genome-wide impact of ecological selection during early stages of speciation-with-gene-flow. *Ecol Lett* **18**, 817-825, doi: 10.1111/ele.12460 (2015).

3 Assour, L. A., LaRosa, N. & Emrich, S. J. Hot RAD: a tool for analysis of next-gen RAD tag data. *arXiv*, 1511.06754 (2015).

4 Dowle, E. J. *et al.* Reproductive isolation and environmental adaptation shape the phylogeography of mountain pine beetle (*Dendroctonus ponderosae*). *Mol Ecol* **26**, 6071-6084, doi: 10.1111/mec.14342 (2017).

5 Catchen, J., Hohenlohe, P. A., Bassham, S., Amores, A. & Cresko, W. A. Stacks: an analysis tool set for population genomics. *Mol Ecol* **22**, 3124-3140, doi: 10.1111/mec.12354 (2013).

6 Catchen, J. M., Amores, A., Hohenlohe, P., Cresko, W. & Postlethwait, J. H. Stacks: building and genotyping loci de novo from short-read sequences. *G3-Genes Genom Genet* **1**, 171-182, doi: 10.1534/g3.111.000240 (2011).

7 Korneliussen, T. S., Albrechtsen, A. & Nielsen, R. ANGSD: Analysis of Next Generation Sequencing Data. *Bmc Bioinformatics* **15**, doi: 10.1186/s12859-014-0356-4 (2014).

8 Ewens, W. J. The sampling theory of selectively neutral alleles. *Theor Popul Biol* **3**, 87-112 (1972).

9 Paradis, E. pegas: an R package for population genetics with an integrated-modular approach. *Bioinformatics* **26**, 419-420, doi: 10.1093/bioinformatics/btp696 (2010).

10 Tajima, F. Statistical method for testing the neutral mutation hypothesis by DNA polymorphism. *Genetics* **123**, 585-595 (1989).

11 Archer, F. I., Adams, P. E. & Schneiders, B. B. stratag: An r package for manipulating, summarizing and analysing population genetic data. *Mol Ecol Resour* **17**, 5-11, doi: 10.1111/1755-0998.12559 (2017).

12 Kamvar, Z. N., Brooks, J. C. & Grunwald, N. J. Novel R tools for analysis of genome-wide population genetic data with emphasis on clonality. *Front Genet* **6**, doi: 10.3389/fgene.2015.00208 (2015).

13 Kamvar, Z. N., Tabima, J. F. & Grunwald, N. J. Poppr: an R package for genetic analysis of populations with clonal, partially clonal, and/or sexual reproduction. *Peerj* **2**, doi: 10.7717/peerj.281 (2014).

14 Cornuet, J. M. *et al.* DIYABC v2.0: a software to make approximate Bayesian computation inferences about population history using single nucleotide polymorphism, DNA sequence and microsatellite data. *Bioinformatics* **30**, 1187-1189, doi: 10.1093/bioinformatics/btt763 (2014).

15 Goudet, J. HIERFSTAT, a package for R to compute and test hierarchical F-statistics. *Mol Ecol Notes* **5**, 184-186, doi: 10.1111/j.1471-8278.2004.00828.x (2005).

16 Evanno, G., Regnaut, S. & Goudet, J. Detecting the number of clusters of individuals using the software STRUCTURE: a simulation study. *Mol Ecol* **14**, 2611-2620, doi: 10.1111/j.1365-294X.2005.02553.x (2005).
